# Patient satisfaction with GP-led melanoma follow-up: a randomised controlled trial

**DOI:** 10.1038/sj.bjc.6605638

**Published:** 2010-05-11

**Authors:** P Murchie, M C Nicolson, P C Hannaford, E A Raja, A J Lee, N C Campbell

**Affiliations:** 1 Centre of Academic Primary Care, University of Aberdeen, Foresterhill Health Centre, Westburn Road, Aberdeen AB25 2AY, UK; 2 ANCHOR Unit, Aberdeen Royal Infirmary, Foresterhill, Foresterhill Road, Aberdeen AB25 2ZD, UK; 3 Section of Population Health, University of Aberdeen, Polwarth Building, Aberdeen, AB25 2AZ, UK

**Keywords:** primary healthcare, cutaneous melanoma, aftercare, survivorship, follow-up

## Abstract

**Background:**

There are no universally accepted guidelines for the follow-up of individuals with cutaneous melanoma. Furthermore, to date, there have been no randomised controlled trials of different models of melanoma follow-up care. This randomised controlled trial was conducted to evaluate the effects of GP-led melanoma follow-up on patient satisfaction, follow-up guideline compliance, anxiety and depression, as well as health status.

**Methods:**

A randomised controlled trial of GP-led follow-up of cutaneous melanoma was conducted over a period of 1 year with assessment by self-completed questionnaires and review of general practice-held medical records at baseline and 12 months later. It took place in 35 general practices in North-east Scotland. Subjects were 142 individuals (51.4% women 48.6% men; mean (s.d.) age 59.2 (15.2) years previously treated for cutaneous melanoma and free of recurrent disease. The intervention consisted of protocol-driven melanoma reviews in primary care, conducted by trained GPs and supported by centralised recall, rapid access pathway to secondary care and a patient information booklet. The main outcome measure was patient satisfaction measured by questionnaire. Secondary outcomes were adherence to guidelines, health status measured by Short Form-36 and the Hospital Anxiety and Depression Scale.

**Results:**

There were significant improvements in 5 out of 15 aspects of patient satisfaction during the study year in those receiving GP-led melanoma follow-up (all *P*⩽0.01). The intervention group was significantly more satisfied with 7 out of 15 aspects of care at follow-up after adjustment for potential confounders. There was significantly greater adherence to guidelines in the intervention group during the study year. There was no significant difference in health status or anxiety and depression between intervention and control groups at either baseline or outcome.

**Conclusions:**

GP-led follow-up is feasible, engenders greater satisfaction in those patients who receive it, permits closer adherence to guidelines and does not result in adverse effects on health status or anxiety and depression when compared with traditional hospital-based follow-up for melanoma.

Patients treated for cutaneous melanoma are at a high risk of recurrence and require careful follow-up (McCarthy *et al*, 1988; Martini *et al*, 1994; Roberts *et al*, 2002; SIGN, 2003). Several guidelines that give recommendations about the timing and schedule of formal follow-up exist, based on current limited evidence and expert opinion (Poo-Hwu *et al*, 1999; Roberts *et al*, 2002; SIGN, 2003). Despite this, there are no universally accepted guidelines for follow-up, and none is specific about where it should take place, or which health-care professional should deliver it (Poo-Hwu *et al*, 1999; Roberts *et al*, 2002; SIGN, 2003). Furthermore, there have been no randomised trials of different follow-up methods, for example, comparing follow-up by different professionals or in different locations (Martinez and Otley, 2001; SIGN, 2003; Francken *et al*, 2005).

There are several reasons to believe that a GP-led follow-up programme for melanoma could be feasible and attractive, but it could also have several potential problems. Individual practices are likely to have small numbers of patients requiring melanoma follow-up, which has the advantage that the programme may be incorporated into the primary care workload with minimal disruption and few resource issues, but the disadvantage that skills might be practised infrequently. However, as melanoma follow-up is based on elements of clinical examination practised routinely in primary care, patient self-examination and patient education, GPs might be able to provide a service that is equivalent in clinical outcome to specialist follow-up. Support for this view comes from three randomised controlled trials, two in women with breast cancer and one in individuals with colorectal cancer, wherein a follow-up programme based on GP examination, patient self-examination and education, demonstrated equivalent outcomes to those achieved in secondary care (Grunfeld *et al*, 1999, 2006; Wattchow *et al*, 2006). Although hospitals may offer more focused specialist consultations, primary care may offer more holistic consultations; this notion has been suggested by earlier studies and was confirmed in a parallel qualitative study to this randomised trial (Grunfeld *et al*, 1999; Murchie *et al*, 2010). A key aim of follow-up is to detect recurrences and new primaries early, but most of these events have been found to occur during the intervals between follow-up appointments; therefore, patient education may be more important than review examinations (Meyers *et al*, 2009). It is not clear whether specialists or primary care are best at enabling patients to understand self-examination, but primary care may have more time, more opportunities for reinforcement and greater continuity. On the other hand, individuals with melanoma may value regular review by specialists and be less reassured by GP review.

We developed a GP-led follow-up programme for cutaneous melanoma according to the principles of the MRC framework for the design and evaluation of complex health-care interventions to improve health (Murchie *et al*, 2007). In a 1-year exploratory randomised controlled trial in North-east Scotland, we evaluated its effect on patient satisfaction, follow-up guideline compliance, anxiety and depression, as well as health status.

## Methods

### Setting and participants

This study was conducted in North-east Scotland (population ∼500 000) between April 2005 and April 2006. A total of 35 general practices from across the region agreed to participate. Each participating practice was presented with a list of their patients currently attending the joint hospital melanoma clinic at the Aberdeen Royal Infirmary (ARI) and asked to supplement this with a search of their own practice computer records for patients with melanoma. They were then asked to ensure that all identified patients met the inclusion criteria (Box 1).

### Sample size calculation

This was an open cluster randomised trial designed to avoid the risk of contamination and minimise bias. As most participants would normally be attending the joint melanoma clinic at the ARI, the size of the intra-cluster correlation coefficient (ICC) between participants at different general practices in baseline satisfaction with existing hospital-based follow-up was unlikely to be large; therefore, a conservative ICC of 0.01 was estimated. With 40 practices and 150 participants, and assuming baseline levels of satisfaction of 60% for service delivery, 65% for the consultation and 40% for continuity of care (Grunfeld *et al*, 1999), the resultant trial would have 80% power to detect differences of at least 20% in each of the three domains of patient satisfaction, at the two-sided 5% significance level.

### Randomisation

Randomisation was conducted by the principal investigator PM. Practices were stratified according to the number of eligible patients likely to be available in each practice on the basis of a list provided by the joint melanoma clinic at the ARI. Three strata were formed: practices with >5 eligible patients, 4–5 eligible patients and ⩽3 eligible participants. Strata were checked to ensure an equal balance of rural (⩾15 miles from the Aberdeen City centre) and urban (<15 miles from the Aberdeen City centre) practices. Practices were then randomised within each stratum to intervention or control using the randomisation function of the computer software package SPSS version 15.0 (SPSS Inc., Chicago, IL, USA). Participants were not blinded as to group allocation.

### The intervention

Complete details of the intervention and its design have been published previously (Murchie *et al*, 2007). Briefly, a lead GP from each of the intervention practices attended a 4-h training session and received a comprehensive information manual detailing how to deliver the study protocol for a 12-month period. This session focused on the presentation of new and recurrent melanomas and how best to examine to identify these. Participating GPs received travel and locum expenses to attend training. In three cases (in which the lead GP was unable to attend the training session), the principal investigator (PM) visited the practices and delivered a modified training session to the GP. All patients in the intervention group received a detailed information booklet about melanoma, which included information on conducting self-examination. A computerised recall register of the intervention group patients was constructed in Microsoft Excel (Microsoft, Redmond, WA, USA) and maintained by the principal investigator. The intervention group patients were invited to attend scheduled protocol-based melanoma follow-up appointments with the lead GP at their practice, at 3 or 6-monthly intervals depending on the thickness of melanoma and time since diagnosis. This schedule of visits is identical to that followed by the specialists running the Joint Melanoma Clinic at the ARI, and is informed by the frequency of visits recommended in national guidelines (Roberts *et al*, 2002; SIGN, 2003). At each appointment, the GP took a focused history, and conducted a structured examination of the melanoma primary site and regional lymph nodes. Imaging, blood tests or dermoscopy were not performed as these are not recommended in the current UK Guidelines (Roberts *et al*, 2002; SIGN, 2003). At the first GP follow-up appointment only, the GP conducted a complete skin survey. Moreover, at the first visit, the GP instructed the patient on how to conduct a self-examination, an issue revisited at each subsequent follow-up visit. Whenever concern arose regarding possible recurrence during or between scheduled follow-up appointments, patients could be referred through a rapid referral pathway to the plastic surgery outpatient clinic at the ARI. Participating practices received £30 per follow-up visit towards practice participation costs. The GPs at control practices received no training in melanoma follow-up and had no scheduled consultations with their patients as part of the study. Patients at practices randomised to the control group continued to attend the hospital-based joint melanoma clinic for their melanoma follow-up, at 3 or 6-monthly intervals depending on the thickness of melanoma and time since diagnosis. The joint melanoma clinic is run by specialists from plastic surgery, oncology and dermatology, and attending patients have primary tumour site, nodal basins, abdomen and other abnormal moles examined by one of these specialists or a trainee.

### Data collection

Data were collected at baseline and after 12 months from the following two sources: (i) a self-completed postal patient questionnaire (comprising questions on demographic and socioeconomic status, a questionnaire to ascertain patient satisfaction (Grunfeld *et al*, 1999), the Short Form-36 (SF-36) questionnaire (Ware, 1993) and the Hospital Anxiety and Depression Scale (HADS) (Zigmond and Snaith, 1983)) and (ii) patients’ general practice-held medical records for information about the initial melanoma diagnosis as well as treatment and use of primary and secondary care services in the previous 12 months.

### Outcome measures

#### Primary outcome

The primary outcome of the trial was patient satisfaction. Patients were asked to rate, on a four-point Likert scale, their agreement with statements about 15 aspects of follow-up covering service delivery, the consultation and continuity of care (Table 2). Owing to small numbers, the answers were combined to create a binary variable for analysis. For each item, ‘agree’ was combined with ‘agree sometimes’ and ‘disagree’ was combined with ‘can’t say’. This was because the satisfaction instrument is attitudinal and based on a Rasch model, in which combing adjacent categories is permitted, provided they signify related levels of the variable being measured. (Wright and Linacre, 1992). The satisfaction questionnaire had been developed for use in a randomised trial of GP-led breast cancer follow-up (Grunfeld *et al*, 1999), and Cronbach's *α-*values of 0.70, 0.67 and 0.70 at baseline, 6 weeks and 3 months have been reported. Answers to the 15 individual questions were then combined into a total satisfaction summary score of between 15 (total satisfaction) and 60 (total dissatisfaction).

#### Secondary outcomes

Adherence to local guidelines was determined in relation to the current locally recommended schedule from the hospital joint melanoma clinic (Box 2). Guideline adherence was a binary variable, with the care of patients considered adherent if the general practice-held medical records indicated that they had had, in the preceding 12 months, the correct number of joint melanoma clinic or GP melanoma follow-up visits as deemed appropriate for the Breslow thickness and time since initial diagnosis. Health status was assessed using the SF-36 (Ware, 1993; Lyons *et al*, 1995) and the HADS (Zigmond and Snaith, 1983; Bjelland *et al*, 2002).

### Statistical analysis

The questionnaires were coded and all data entered manually using Microsoft Office Access 2003 (Microsoft). The data were analysed using SPSS for Windows version 15.0 and STATA 10 (StataCorp, College Station, TX, USA).

Satisfaction, adherence to guidelines and HADS status between intervention and control groups at baseline and follow-up were compared using the cluster-adjusted *χ*^2^ test based on the generalised estimating equations (GEEs) approach, on the assumption that there was working exchangeable correlation among patients within the same cluster (Donner and Klar, 1994). It is now recognised that changes in SF-36 scores between two points in time for six domains (namely physical functioning, social functioning, mental health, energy and vitality, bodily pain and general health), as well as for the physical and mental component summary scores have a normal distribution (Fayers and Machin, 2000). For this reason, the cluster-adjusted *t*-test was used to compare the mean summary satisfaction score and SF-36 scores between intervention and control groups at the two time points (Donner and Klar, 2000). The cluster-adjusted McNemar's *χ*^2^ test was used to compare change in each aspect of satisfaction, adherence to guidelines and HADS group between baseline and follow-up within the intervention and control groups separately (Durkalski *et al*, 2003). Unadjusted odds ratios (ORs) and their 95% confidence interval (95% CI) were estimated through GEEs using robust (heteroskedasticity-corrected) s.e. to increase the efficiency of estimation (Donner and Klar, 1994). The ORs were then adjusted for variables selected *a priori* as potential confounders (gender, age, practice size, distance of home from ARI, educational status, level of satisfaction at baseline and practice). Owing to the number of statistical tests performed, and the increased chance of a type 1 error, a *P*-value of ⩽0.01 was used to denote statistical significance throughout the univariate analyses.

## Results

### Recruitment

**Figure 1:**
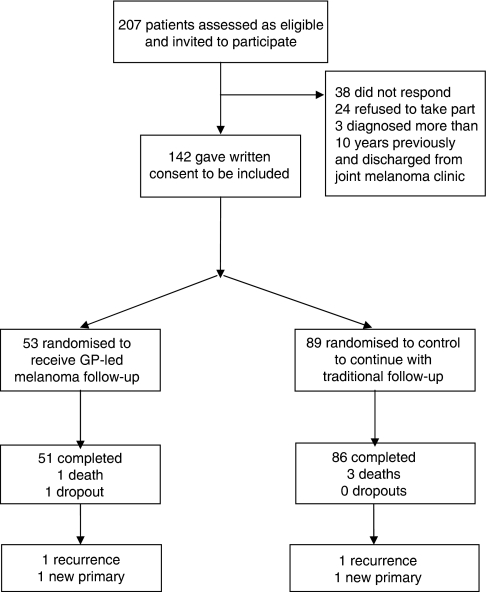
Consort diagram illustrating participant flow.

Overall, 35 of the 83 practices approached (42.2%) agreed to participate, and a total of 207 potentially eligible patients were identified (Figure 1). Of these, 142 (68.6%) patients agreed to participate in the study, resulting in 17 practices with 53 patients in the intervention group and 18 practices with 89 patients in the control group. The imbalance in numbers was a result of lower recruitment than anticipated in several larger practices.

The intervention and control groups were comparable for age, sex, socioeconomic factors and features of their melanoma, but those in the intervention group tended to live further away from the ARI (Table 1).

### Patient satisfaction

At baseline, there was no significant difference between groups in any of the 15 aspects of the patient satisfaction measured (Table 2). At follow-up, more participants in the intervention group agreed with different aspects of satisfaction than did those in the control group. There were statistically significant differences between groups on 6 of the 15 aspects assessed. Members of the intervention group were significantly more likely than those in the control group to believe that ‘it's easy to get through by phone if you need to’ that ‘if I feel I need to I can usually see a doctor on the same day’ that ‘you are usually seen within 20 minutes of your appointment time’ that ‘the doctor examines you thoroughly when necessary’ that ‘the doctor always prescribes medicine if you need it’ and that ‘you see a doctor who know you well.’

At baseline, there was no significant difference between groups in the cluster-adjusted mean summary satisfaction score (*P*=0.912). At follow-up, the cluster-adjusted mean score was significantly lower in the intervention group 26.4 (95% CI: 24.9–27.9) *vs* 33.5 (95% CI: 32.5–34.4), indicating significantly higher satisfaction than in controls (*P*<0.001).

Within the intervention group, there was a statistically significant change over time in the level of agreement in satisfaction with 5 of 15 aspects of care (Table 3). In contrast, within the control group, there were no significant changes over time. Change (from baseline to follow-up) in the mean summary score was found to be significantly lower (−5.96, 95% CI: −8.09 to −3.89) in the intervention group than in the control group (0.29, 95% CI: −1.49 to 2.08) (*P*=0.001), indicating higher overall satisfaction.

Of the 13 aspects of satisfaction wherein ORs could be computed, there was statistically significant higher satisfaction in the intervention group for 7 aspects after adjustment for clustering and potential confounders (Table 4).

### Adherence to guidelines

In the year before the study, 84.9% of the individuals in the intervention group and 85.4% of the control group had been seen in accordance with the local guidelines (*P*=0.86). At follow-up, 98.1% of the individuals in the intervention group were seen according to local guidelines, compared with 80.9% of the control group (*P*=0.020).

### Health status and psychological well-being

There was no statistically significant difference between groups in terms of distribution of SF-36 scores at either baseline or follow-up (Table 5). There was no statistically significant difference in the proportion of intervention and control group participants with a HADS score, suggesting anxiety or depression (⩾8) either at baseline or follow-up (Table 6). Similarly, there was no statistically significant within-group difference between baseline and follow-up in the proportion of patients exhibiting a likelihood of caseness for anxiety or depression (HADS score ⩾8) (Table 7) (Bjelland *et al*, 2002).

### Referrals and adverse events

A total of 146 GP follow-up appointments took place during the study year. No problems were detected during 136 (93.2%) of these appointments. In all, 10 (6.8%) scheduled follow-up appointments resulted in urgent referrals through the rapid access pathway.

Nine adverse events occurred during the trial, of which four were related to melanoma. There were two diagnoses of recurrent melanoma, one in each group, with one patient dying before the study was completed. Two new primaries, one in each group, were detected during the trial year. Unrelated to melanoma, three patients died (all belonging to the control group) and two, one in either group, were diagnosed with another cancer.

## Discussion

### Main findings

We found that GP-led melanoma follow-up improved patient satisfaction and was more guideline compliant than hospital-based follow-up. Health status and psychological well-being of those participants receiving the new intervention seemed to be relatively unaffected.

### Strengths and limitations

The GP-led follow-up programme was developed over a period of 12 months using the MRC framework for the development and evaluation of complex interventions to improve health (Murchie *et al*, 2007), resulting in a robust intervention that functioned with few practical difficulties. A total of 42% of practices in the Grampian region agreed to participate in the study. This is a relatively high recruitment rate for an interventional trial in primary care and increases the chances that the results are applicable to the whole of this large geographical area (Foy *et al*, 2003). We randomised by practice to avoid contamination, but lower than expected recruitment in some practices resulted in eight clusters with fewer than three participants each after follow-up. This complicated our analysis, but the use of the GEE approach enabled us to account for the effect of clustering throughout all analyses.

We were unable to investigate the effect of the intervention on clinical end points, such as recurrence and mortality. *Post hoc* power calculations indicate that, to obtain definitive data on mortality and recurrence, a study of at least 2000 patients would be required, and this is unlikely ever to be funded. Our study, therefore, provides a firm basis if such a major undertaking were ever to be contemplated. On the other hand, it seems likely that research in this important clinical area will have to depend in future on softer outcomes, and our data contribute considerably to sparse current literature. Furthermore, although we detected no significant difference in health status or anxiety and depression, our trial had limited power to detect small or moderate-sized changes.

The trial was not blinded. Patients were aware that they were in the control group, not receiving the novel GP-led follow-up programme. Our results would have been affected if patients in the control group became, or remained, more dissatisfied than those in the intervention group, because they knew that they remained under hospital follow-up.

Our recruitment rate of 67% is high, but 38 of those patients invited to participate did not respond and 28 declined to take part. We have no information on the reasons for refusal or non-response, but it may be that GP-led melanoma follow-up is attractive and acceptable to many, but not all, patients.

Owing to limitations of funding, the study ran for only 1 year. According to the protocol, 22 members of the intervention group required a 3-monthly follow-up and 31 required a 6-monthly follow-up. As a result, the median number of GP-led melanoma follow-up appointments experienced by the intervention group was two. This may have limited their ability to make comparisons with their hospital follow-up. At the conclusion of the trial, melanoma follow-up by GPs ended and patients were discharged back to the melanoma clinic. Although the intervention seemed to have been successful over the course of the year, it is not known whether the benefits would have been sustained over a longer period.

### Comparison with other research

Several questionnaire and qualitative studies have explored the views of patients about the possibility of receiving cancer follow-up from their GPs (Adewuyi-Dalton *et al*, 1998; Howells *et al*, 1999; Rozmovits *et al*, 2004; Dancey *et al*, 2005). Most suggest that a significant proportion of patients would consider primary care-led cancer follow-up to be acceptable, including 60% of 217 individuals attending follow-up after melanoma at a plastic surgical outpatient clinic in an English hospital in 2003 (Dancey *et al*, 2005). Three randomised trials, two for breast and one for colorectal cancer, have examined follow-up based in primary care, with the GP responsible for delivering the intervention (Grunfeld *et al*, 1996, 1999, 2006; Wattchow *et al*, 2006). In each case, the GP-led programme performed at least as well as traditional hospital-based follow-up, although the outcome measures varied from trial to trial. Several small surveys provide evidence about the willingness of UK GPs to become involved in the follow-up of patients with cancer, including melanoma (Grunfeld *et al*, 1995; Papagrigoriadis and Heyman, 2001; Moses *et al*, 2004; Dancey *et al*, 2005).

Taken together, the evidence suggests that a sizeable proportion of individuals with cancer are willing to consider alternatives to traditional hospital follow-up. In addition, it is clear that groups of health professionals, including GPs and specialist nurses, are willing, with certain caveats, to assume greater responsibility for the routine follow-up of patients with cancer (Grunfeld *et al*, 1995; Papagrigoriadis and Heyman, 2001; Moses *et al*, 2004; Cox *et al*, 2006). Our results seem to strengthen these findings, as they support the notion that GP-led follow-up would be attractive to a large proportion of individuals with melanoma, and their GPs. It is important to be aware, however, that this is just one alternative model for the delivery of cancer follow-up care.

### Meaning and implications

Our results show that an integrated programme of routine GP-led follow-up for patients with cutaneous melanoma is feasible, at least in North-east Scotland. Receipt of follow-up in general practice, instead of at the hospital outpatient clinic, did not significantly increase anxiety or depression among participants, or impair their health status, although, as these were secondary outcomes, we had limited power to state this definitively. These, although limited, data are consistent with earlier work suggesting that most recurrent cancers are detected in the interval between routine follow-up appointments. Overall, the data support an emerging literature which suggests that GP-led follow-up confers important benefits in patient satisfaction, without adverse effects on the psychological and physical well-being of patients, at least in the short term. This finding needs to be confirmed in large-scale randomised trials powered on hard clinical outcomes, such as recurrence and mortality.

## Figures and Tables

**Table 1:** Baseline characteristics of each group

	**Intervention**	**Control**
*Demographics*		
Gender		
Male *n* (%)	28 (52.8)	41 (46.1)
Female *n* (%)	25 (47.2)	48 (53.9)
Age at start of trial		
Years – mean (s.d.)	58.7 (14.6)	59.5 (15.5)
Employment status		
Full-time work *n* (%)	21 (42.0)	30 (38.0)
Part-time work *n* (%)	4 (8.0)	9 (11.4)
Retired *n* (%)	24 (48.0)	34 (43.0)
Unemployed *n* (%)	0 (0)	1 (1.3)
At home *n* (%)	1 (2.0)	2 (2.5)
Other *n* (%)	0 (0)	3 (3.8)
Educational level		
None *n* (%)	11 (22)	14 (17.5)
O grades *n* (%)	8 (16.0)	15 (18.8)
Highers *n* (%)	1 (2.0)	3 (3.8)
Vocational or technical *n* (%)	13 (26.0)	13 (16.3)
Degree or professional *n* (%)	17 (34.0)	33 (41.3)
Other *n* (%)	0 (0)	2 (2.5)
		
*Melanoma details*		
Site		
Head or neck *n* (%)	13 (24.5)	15 (16.9)
Back *n* (%)	9 (17.0)	17 (19.1)
Chest or abdomen *n* (%)	8 (15.1)	7 (7.9)
Arm *n* (%)	5 (9.4)	11 (12.4)
Leg *n* (%)	15 (28.3)	28 (31.5)
Acral *n* (%)^a^	2 (3.8)	11 (12.4)
Primary not identified *n* (%)	1 (1.9)	0 (0)
Breslow thickness		
Median (IQR)	0.6 (0.25–1.5)	0.9 (0.4–1.7)
Breslow thickness categories (mm)		
<1 *n* (%)	30 (56.6)	48 (53.9)
1–4 *n* (%)	20 (37.7)	33 (37.1)
>4 *n* (%)	3 (5.7)	8 (9.0)
Time since diagnosis at the start of the trial (months)		
Median (IQR)	49 (19–76)	63 (31–88.5)
Time since diagnosis categories (months)		
1–36 *n* (%)	23 (43.4)	28 (31.5)
37–120 *n* (%)	23 (43.4)	55 (61.8)
121–316 *n* (%)	7 (13.2)	6 (6.7)
		
*Home location*		
Distance from ARI		
Miles—median (IQR)	27.6 (18.9–32.3)	10.1 (2.3–25.9)
Distance from GP practice		
Miles—median (IQR)	1.6 (0.8–5.0)	1.5 (0.8–3.6)
Distance categories (miles)		
0–19 *n* (%)	15 (28.3)	60 (67.4)
20–39 *n* (%)	31 (68.9)	14 (15.7)
>40 *n* (%)	7 (31.8)	15 (16.9)
Travelling time to ARI		
Minutes—median (IQR)	38.0 (28.5–46.0)	19.0 (7.0–33.0)
Travelling time categories (min)		
0–30 *n* (%)	15 (28.3)	63 (70.8)
31–60 *n* (%)	32 (60.4)	14 (15.7)
60–90 *n* (%)	6 (11.3)	12 (13.5)
		
*Practice*		
Practice size (list size at participants general practice)		
Up to 7000 *n* (%)	4 (7.5)	14 (15.7)
7000–10 000 *n* (%)	31 (58.5)	23 (25.8)
More than 10 000 *n* (%)	18 (34.0)	52 (58.4)
		
*Follow-up attendance*		
Total joint melanoma clinic attendances before trial		
Median (IQR)	6 (2–11)	8 (2–12)
Joint melanoma clinic attendance categories		
0 *n* (%)	11 (20.8)	18 (20.2)
1–6 *n* (%)	16 (30.2)	20 (22.5)
7–26 *n* (%)	26 (49.1)	51 (57.3)

Abbreviations: IQR=interquartile range; ARI=Aberdeen Royal Infirmary; GP=general practitioner.

a Acral melanomas are those affecting the extremities, namely nail bed, palmar and plantar melanomas.

**Table 2:** Proportion of people indicating satisfaction with different aspects of care at baseline and follow-up, by group

		**Agree *n* (%)**	**Do not agree *n* (%)**		**Agree *n* (%)**	**Do not agree *n* (%)**	
**Aspect of satisfaction**	**Group**	**Baseline**	**Baseline**	***P*-value**	**Follow-up**	**Follow-up**	***P*-value**
*Service delivery*							
It is easy to get through by phone if you need to	Intervention	33 (70.2)	14 (29.8)		48 (98.0)	1 (2.0)	
	Control	52 (73.2)	19 (26.8)	0.799	49 (71.0)	20 (29.0)	**<0.001**
The reception staff are helpful	Intervention	47 (97.9)	1 (2.1)		48 (98.0)	1 (2.0)	
	Control	71 (97.3)	2 (2.7)	0.955	62 (91.2)	6 (8.8)	0.112
If I feel I need to I can usually see a doctor on the same day	Intervention	17 (36.2)	30 (63.8)		40 (81.6)	9 (18.4)	
	Control	22 (31.0)	49 (69.0)	0.512	26 (38.8)	41 (61.2)	**<0.001**
You are usually seen within 20 min of your appointment time	Intervention	37 (75.5)	12 (24.5)		49 (100)	0 (0)	
	Control	56 (75.7)	18 (24.3)	0.991	54 (78.3)	15 (12.7)	**<0.001**
							
*The consultation*							
There is not enough time to discuss your problems with the doctor	Intervention	20 (41.7)	28 (58.3)		9 (18.4)	40 (81.6)	
	Control	29 (40.8)	42 (59.2)	0.899	21 (30.9)	47 (69.1)	0.064
You get good advice about how to keep yourself healthy	Intervention	32 (68.1)	15 (31.9)		46 (93.9)	3 (6.1)	
	Control	51 (70.8)	21 (29.2)	0.738	53 (77.9)	15 (22.1)	0.022
It is sometimes difficult to discuss your concerns with the doctor	Intervention	22 (45.8)	26 (54.2)		8 (16.3)	41 (83.7)	
	Control	20 (28.2)	51 (71.8)	0.087	17 (25.4)	50 (74.6)	0.371
The doctor explains clearly what is wrong	Intervention	40 (87.0)	6 (13.0)		46 (95.8)	2 (4.2)	
	Control	60 (84.5)	11 (15.5)	0.957	62 (91.2)	6 (8.8)	0.330
The doctor examines you thoroughly when necessary	Intervention	44 (89.8)	5 (10.2)		49 (100)	0 (0)	
	Control	65 (90.3)	7 (9.7)	0.957	61 (91.0)	6 (9.0)	**<0.001**
Sometimes you feel the doctor should listen more to what you say	Intervention	19 (39.6)	29 (60.4)		9 (18.8)	39 (81.3)	
	Control	18 (25.7)	52 (74.3)	0.146	15 (22.7)	51 (77.3)	0.664
The doctor should tell you more about your problem and treatment	Intervention	27 (57.4)	20 (38.5)		13 (27.1)	35 (72.9)	
	Control	38 (54.3)	32 (45.7)	0.649	20 (30.8)	45 (69.2)	0.338
The doctor encourages you to talk about your problem and treatment	Intervention	31 (66.0)	16 (34.0)		45 (91.8)	4 (8.2)	
	Control	58 (81.7)	13 (18.3)	0.039	52 (78.8)	14 (21.2)	0.108
The doctor always prescribes medicine if you need it	Intervention	15 (30)	35 (70)		38 (77.6)	11 (22.4)	
	Control	17 (24.6)	52 (75.3)	0.501	19 (28.8)	47 (71.2)	**<0.001**
							
*Continuity of care*							
You see a doctor who knows you well	Intervention	19 (38.8)	30 (61.2)		45 (91.8)	4 (8.2)	
	Control	30 (41.1)	43 (58.9)	0.784	33 (48.5)	35 (51.5)	**<0.001**
If you need to see a doctor, you have to wait too long for the doctor you want	Intervention	11 (22.4)	38 (77.6)		14 (28.6)	35 (71.4)	
	Control	15 (21.7)	54 (78.3)	0.884	15 (22.4)	52 (77.6)	
							
		**Baseline**			**Intervention**		
*Satisfaction summary score* a	Intervention	32.6 (30.4–34.8)			26.4 (24.9–27.9)		
	Control	32.5 (30.7–34.2)		0.912	33.5 (32.5–34.4)		**<0.001**

a Values are mean (95% confidence interval). The bolded *P* values indicate those associated with significance differences.

**Table 3:** Number of people indicating satisfaction with different aspects of care before and after the intervention

		**Intervention**			**Control**		
		**Follow-up**			**Follow-up**		
**Aspect of satisfaction**	**Baseline**	**Agree *n***	**Do not agree *n***	***P*-value**	**Agree *n***	**Do not agree *n***	***P*-value**
*Service delivery*							
It is easy to get through by phone if you need to	Agree	30	1		38	6	
	Disagree	14	0	0.022	8	10	0.231
The reception staff are helpful	Agree	44	1		58	3	
	Disagree	1	0	0.654	1	1	0.516
If I feel I need to I can usually see a doctor on the same day	Agree	14	3		16	4	
	Disagree	22	6	0.003	9	31	0.230
You are usually seen within 20 min of your appointment time	Agree	36	0		45	4	
	Disagree	11	0	0.017	8	8	0.298
							
*The consultation*							
There is not enough time to discuss your problems with the doctor	Agree	5	13		13	11	
	Disagree	3	25	0.014	6	31	0.321
You get good advice about how to keep yourself healthy	Agree	31	0		40	6	
	Disagree	12	2	0.005	11	6	0.640
It is sometimes difficult to discuss your concerns with the doctor	Agree	4	16		8	6	
	Disagree	3	23	0.011	7	41	0.921
The doctor explains clearly what is wrong	Agree	37	0		51	2	
	Disagree	5	1	0.056	7	2	0.046
The doctor examines you thoroughly when necessary	Agree	43	0		54	2	
	Disagree	4	0	0.162	4	2	0.310
Sometimes you feel the doctor should listen more to what you say	Agree	4	14		6	7	
	Disagree	4	23	0.019	7	40	0.922
The doctor should tell you more about your problem and treatment	Agree	11	14		14	14	
	Disagree	1	18	0.008	2	29	0.012
The doctor encourages you to talk about your problem and treatment	Agree	29	0		46	5	
	Disagree	13	3	0.014	3	7	0.313
The doctor always prescribes medicine if you need it	Agree	13	2		11	4	
	Disagree	24	9	0.001	6	38	0.311
							
*Continuity of care*							
You see a doctor who knows you well	Agree	18	0		21	3	
	Disagree	25	4	<0.001	11	29	0.074
If you need to see a doctor, you have to wait too long for the doctor you want	Agree	5	5		7	5	
	Disagree	8	29	0.510	7	41	0.723

**Table 4:** Chances of agreeing at follow-up with various statements of satisfaction with aspects of care of follow-up in the intervention group, compared with the control group (expressed as odds ratio (95% confidence interval (CI)

**Aspect of patient satisfaction**	**Unadjusted odds ratio (95% CI)**	**Adjusted odds ratio^a^ (95%CI)**
*Service delivery*		
It is easy to get through by phone if you need to	18.0 (2.5–128.6)	26.3 (4.8–143.2)
The reception staff are helpful	5.1 (0.7–38.5)	5.4 (1.0–28.5)
If I feel I need to I can usually see a doctor on the same day	6.7 (2.6–17.1)	7.6 (2.1–26.9)
You are usually seen within 20 min of your appointment time	NA	NA
		
*The consultation*		
There is not enough time to discuss your problems with the doctor	0.5 (0.2–1.0)	0.4 (0.2–0.8)
You get good advice about how to keep yourself healthy	4.3 (1.2–14.9)	8.1 (1.2–53.9)
It is sometimes difficult to discuss your concerns with the doctor	0.6 (0.2–2.0)	0.3 (0.1–1.1)
The doctor explains clearly what is wrong	2.3 (0.4–11.5)	25.8 (2.0–332.6)
The doctor examines you thoroughly when necessary	NA	NA
Sometimes you feel the doctor should listen more to what you say	0.9 (0.4–1.7)	1.3 (0.6–2.9)
The doctor should tell you more about your problem and treatment	0.8 (0.6–1.3)	0.7 (0.3–1.4)
The doctor encourages you to talk about your problem and treatment	2.9 (0.8–10.9)	88.7 (7.2–1087.1)
The doctor always prescribes medicine if you need it	8.7 (3.4–22.5)	23.8 (6.0–95.1)
		
*Continuity of care*		
You see a doctor who knows you well	12.5 (4.6–33.8)	30.2 (8.5–107.8)
If you need to see a doctor, you have to wait too long for the doctor you want	1.4 (0.6–2.9)	0.8 (0.3–1.8)

Abbreviations: CI=confidence interval; NA=not applicable.

These odds ratios could not be computed because of the small number of data and the consequent instability of the model.

a Adjusted for gender, age, practice size, distance of home from the Aberdeen Royal Infirmary, educational status and level of agreement at baseline.

**Table 5:** Median and interquartile range (IQR) SF-36 scores

			**Median**	**IQR**	***P*-value**
Physical functioning	Baseline	Intervention	95	90–100	
		Control	90	80–100	0.273
	Outcome	Intervention	95	85–100	
		Control	90	70.55–100	0.330
Social functioning	Baseline	Intervention	100	75–100	
		Control	100	75–100	0.344
	Outcome	Intervention	100	78.125–100	
		Control	100	75–100	0.560
Role physical	Baseline	Intervention	100	100–100	
		Control	100	75–100	0.381
	Outcome	Intervention	100	100–100	
		Control	100	50–100	0.149
Role emotional	Baseline	Intervention	100	100–100	
		Control	100	100–100	0.467
	Outcome	Intervention	100	100–100	
		Control	100	100–100	0.419
Mental health	Baseline	Intervention	84	68–92	
		Control	82	68–92	0.898
	Outcome	Intervention	84	72–92	
		Control	84	72–92	0.730
Energy and vitality	Baseline	Intervention	67.5	55–83.75	
		Control	70	52–96	0.793
	Outcome	Intervention	70	60–80	
		Control	70	46.25–83.75	0.698
Bodily pain	Baseline	Intervention	74	52–96	
		Control	84	62–100	0.309
	Outcome	Intervention	84	62–100	
		Control	84	62–100	1.000
General health	Baseline	Intervention	77	63.25–97	
		Control	77	67–87	0.772
	Outcome	Intervention	82	67–92	
		Control	77	60–87	0.589

Abbreviations: IQR=interquartile range; SF-36=Short Form-36.

**Table 6:** Baseline and outcome hospital anxiety and depression scale scores

				***P*-value**
Baseline	Intervention	Anxious	11	
		Not anxious	39	
	Control	Anxious	21	
		Not anxious	60	0.611
	Intervention	Depressed	4	
		Not depressed	46	
	Control	Depressed	9	
		Not depressed	72	0.563
				
Outcome	Intervention	Anxious	9	
		Not anxious	39	
	Control	Anxious	13	
		Not anxious	61	0.868
	Intervention	Depressed	3	
		Not depressed	45	
	Control	Depressed	5	
		Not depressed	69	0.912

Figures are number of people with a score of ⩾8 defined as depressed or anxious.

**Table 7:** Comparison of hospital anxiety and depression scale scores within groups between baseline and outcome

	**Intervention**			**Control**		
	**Outcome**			**Outcome**		
**Baseline**	**Anxious**	**Not anxious**	***P*-value**	**Anxious**	**Not anxious**	***P*-value**
Anxious	4	5		7	11	
Not anxious	5	33	1.000	6	49	0.332
	Depressed	Not depressed		Depressed	Not depressed	
Depressed	2	1		2	7	
Not depressed	1	43	1.000	3	61	0.344

Figures are number of people with a score of ⩾8 defined as depressed or anxious.
